# EFFECT OF A 6-MONTH HOME-BASED WALKING PROGRAM ON COGNITIVE FITNESS IN OLDER ADULTS WITH CHRONIC KIDNEY DISEASE

**DOI:** 10.1093/geroni/igac059.1553

**Published:** 2022-12-20

**Authors:** Ulf Bronas

**Affiliations:** University of Illinois Chicago, Chicago, Illinois, United States

## Abstract

We have previously demonstrated that cognitive status and white matter integrity are highly associated with physical fitness in older adult patients with chronic kidney disease (CKD). We present data from a two group RCT determining effect of a 6-month home-based walking intervention on physical and cognitive fitness compared to usual care. The intervention included use of a wearable activity monitor, weekly didactic phone meetings, interactive tools and monthly coach-delivered feedback. The intervention group had a 78% compliance rate to the 6-month exercise program (159.9 (149.2) minutes/week). Executive function composite score and global cognitive for intervention group > than control group. Additionally, global white matter integrity and functional connectivity improved in the intervention group and declined in the usual care group. We conclude that a home-based physical activity intervention can have significant improvement on cognitive health and neuroplasticity in older adults with CKD and lowering their risk of developing AD/ADRD

